# Mutation Screening of Multiple Genes in Spanish Patients with Autosomal Recessive Retinitis Pigmentosa by Targeted Resequencing

**DOI:** 10.1371/journal.pone.0027894

**Published:** 2011-12-02

**Authors:** María González-del Pozo, Salud Borrego, Isabel Barragán, Juan I. Pieras, Javier Santoyo, Nerea Matamala, Belén Naranjo, Joaquín Dopazo, Guillermo Antiñolo

**Affiliations:** 1 Unidad de Gestión Clínica de Genética, Reproducción y Medicina Fetal, Instituto de Biomedicina de Sevilla, Hospital Universitario Virgen del Rocío/CSIC/Universidad de Sevilla, Sevilla, Spain; 2 Centro de Investigación Biomédica en Red de Enfermedades Raras (CIBERER), Sevilla, Spain; 3 Medical Genome Project, Andalusian Center for Human Genomic Sequencing, Sevilla, Spain; 4 Departamento de Bioinformática y Genómica, Centro de Investigación Príncipe Felipe, Valencia, Spain; 5 Functional Genomics Node (INB), Valencia, Spain; Deutsches Krebsforschungszentrum, Germany

## Abstract

Retinitis Pigmentosa (RP) is a heterogeneous group of inherited retinal dystrophies characterised ultimately by the loss of photoreceptor cells. RP is the leading cause of visual loss in individuals younger than 60 years, with a prevalence of about 1 in 4000. The molecular genetic diagnosis of autosomal recessive RP (arRP) is challenging due to the large genetic and clinical heterogeneity. Traditional methods for sequencing arRP genes are often laborious and not easily available and a screening technique that enables the rapid detection of the genetic cause would be very helpful in the clinical practice. The goal of this study was to develop and apply microarray-based resequencing technology capable of detecting both known and novel mutations on a single high-throughput platform. Hence, the coding regions and exon/intron boundaries of 16 arRP genes were resequenced using microarrays in 102 Spanish patients with clinical diagnosis of arRP. All the detected variations were confirmed by direct sequencing and potential pathogenicity was assessed by functional predictions and frequency in controls. For validation purposes 4 positive controls for variants consisting of previously identified changes were hybridized on the array. As a result of the screening, we detected 44 variants, of which 15 are very likely pathogenic detected in 14 arRP families (14%). Finally, the design of this array can easily be transformed in an equivalent diagnostic system based on targeted enrichment followed by next generation sequencing.

## Introduction

Retinitis pigmentosa (RP, OMIM 26800) is a heterogeneous group of inherited retinal dystrophies caused by the progressive loss of photoreceptors. Typically it presents with poor night vision in early or middle life, followed by the constriction of the visual field and progressive loss of visual acuity leading to complete blindness after several decades. On ophthalmic examination, RP manifests with retinal pigmentation, attenuated retinal blood vessels, and waxy optic disc pallor associated with a diminished or abolished electroretinogram. Prevalence of nonsyndromic RP is approximately 1 in 4000 [1]. The condition may segregate as an autosomal dominant RP (24%), autosomal recessive (41%), or an X-linked recessive trait (22%), and the remaining 12% of cases were presumed to result from non-genetic factors, non-Mendelian inheritance (for example mitochondrial or *de novo* mutations) or complex inheritance (digenic or polygenic inheritance) [2].

To date, 37 *loci* have been reported being responsible for autosomal recessive RP (arRP), of which 34 genes have been identified [3]. However, all together the reported *loci* are responsible for only ∼35–45% of the recessive RP cases, none of them independently account for a substantial proportion of arRP cases [4]. Recently, we have identified a new gene as the most common single gene that causes arRP: *Eyes Shut Homologue (EYS*; 15.9% of cases) [5], almost all causal mutations associated with arRP are rare (minor allele frequency (MAF) <<0.01) and most of the associated genes have hundreds of disease alleles with potentially different pathogenic effects [2].

The identification of a causative mutation is important to ascertain the genetic basis of the disease, and thus paves the way for genetic counselling, family planning and future gene-targeted treatment. Nevertheless, further strategies such as establishing a classification of the mutations as loss- or gain-of-function and assessing the functionality of the mutant proteins, should be undertaken to develop a treatment tailored to each of the different arRP genes.

Molecular diagnosis of arRP mutations is challenging because a large number of deleterious mutations can be found in each gene, and multiple genes can be mutated to give the same phenotype. Thus, the extensive genetic and allelic heterogeneity of retinal dystrophies makes mutation detection by current molecular techniques problematic. Pre-screening tools, such as single-strand chain polymorphism and denaturing high-pressure liquid chromatography are economic techniques capable of detecting known changes, but not to identify new ones. Similarly, there are commercial genotyping microarrays available, based on the arrayed primer extension (APEX) technology [6], which enable the simultaneous screening of multiple genes but they can only detect a fixed number of known mutations. However, the extensive genetic heterogeneity along with the still unknown repertoire of arRP mutations requires of the use of a tool that can identify both, known and new mutations, in a large number of genes in a fast manner. Dideoxy sequencing method can identify mutations but its use for the screening of multiple genes is so time-consuming and expensive that is inapplicable in this scenario. Recently, emerging technologies for ultra high throughput sequencing have started to be applied to diagnostic in a prospective manner [7], [8]. However, the use of these technologies for screening a set of disease causing genes is still limited because of the perceived technical and data-handling challenges. Targeted resequencing offers a solution of compromise that can have a practical application in clinics. Nevertheless, the use of a capture system for the enrichment of the target sequences, followed by ultra-high throughput sequencing, is still a complex technology not available in many laboratories and with many problems of standardisation that needs still to be solved. There are, though, alternative approaches that can be used while the new sequencing technologies become applicable in this context. Thus, while the past decade has witnessed the development of sequencing by hybridization to oligonucleotides on an array [9], recently significant improvements have been made in this technology, and resequencing microarrays offer the potential of determining the sequence of a large number of genes with a reasonable amount of effort and cost [10]. Herein, we present the development and validation of a custom design resequencing microarray which allows a widespread screening of both, novel and known mutations, in 16 genes related to arRP. Although this technology has already been used as a diagnostic test to investigate arRP [10]–[13], this is the first report of the application of these resequencing platforms which involves the study of the most prevalent gene, *EYS*. A cohort of 102 arRP patients from Spain was screened with the new RP genechip, demonstrating the potential clinical utility of this technology.

## Methods

### Ethics Statement

The study conformed to the tenets of the declaration of Helsinki (Edinburgh, 2000) and was approved by the Institutional Review Board of the Hospital Virgen del Rocío, Seville. An informed consent form was signed by all participants for clinical and molecular genetic studies.

### Subjects and Clinical Data

The study cohort comprises 102 Spanish unrelated patients affected by arRP. A full ophthalmic examination was performed as described elsewhere [14]. RP was defined as bilateral visual loss, initial hemeralopy, restriction of visual field, gradual increased bone spicule pigmentation and decrease of visual acuity, attenuation of retinal vessels, reduced or undetectable electroretinogram (ERG) and waxy disc pallor. Globally, our cohort included 98 arRP patients with no known mutations and 4 arRP patients with 4 pathogenic variants included as positive controls of mutations, previously identified by dideoxy sequencing (Table 1). In addition, available samples of proband family members were tested for co-segregation studies. A group of 100 control individuals was also recruited which comprised unselected, unrelated race-, age-, and gender-matched individuals from Spain.

**Table 1:** Known Sequence Changes Tested in the Validation and Reproducibility assay.

Family ID	Gene	Nucleotide changeProtein changeGenotype	MutationType	Calls of the arrays using the IUPAC Base Code*	Reference
RP 21	*TULP1*	c.823-4A>GHeterozygous	Splice site mutation	Detected as R	[37]
RP 242	*TULP1*	c.430A>Gp.K96EHeterozygous	Missense	Detected as R	Unpublished
RP 57	*TULP1*	c.1255C>Gp.R419GHeterozygous	Missense	Detected as S	Unpublished
RP 60	*EYS*	c.78-79insGCp.Q27RfsX16Heterozygous	Insertion frameshift	Not Detected	[5]

*IUPAC Base Codes: The symbol R to designate PuRine (A or G); S to designate Strong interaction (C or G).

### Custom Genome Resequencing Microarray Design

The sequences comprising all coding exons plus 15 bp of flanking intronic sequence from the arRP genes *CERKL*, *CNGA1*, *CRB1*, *EYS*, *IDH3B*, *LRAT*, *MERTK*, *NR2E3*, *PDE6B*, *PRCD*, *PROM1*, *RGR*, *RHO*, *RLBP1*, *RPE65* and *TULP1* were selected to tile on the resequencing microarray (Affymetrix, Santa Clara, CA). Repetitive elements and internal duplications that may lead to cross hybridization were identified by using Repeat Masker [15] and deleted. For each position of the interrogated sequence, eight 25-mer probes are represented on the array: four probes for each strand, each with a different nucleotide in the middle (A,G,C,T)—one perfect match for the reference sequence and three mismatches—allowing the detection of all possible nucleotide substitutions of both strands. In total, 45,096 bp features are tiled on the array and 44,282 bp of double-stranded gene sequences are analyzed. The remaining oligonucleotides represent control DNA (Affymetrix control reference sequence; AFFX-TagIQ-EX). Our custom designed arRP chips were fabricated by Affymetrix using standard photolithography and solid-phase DNA synthesis [9], [16], [17].

### Experimental Procedure and Data Analyses

A total of 93 polymerase chain reaction (PCR) amplicons (ranging from 300 bp to 7.5 Kbp) were designed and optimized to amplify under a common set of Short-Range and Long-Range PCR conditions. Primer sequences and PCR conditions employed are available upon request. DNA concentration of each amplicon was measured using a picogreen assay and PCR products were pooled and purified of residual primers and nucleotides using Clontech purification plates (Clontech, Mountain View, CA). The DNA was then fragmented, labeled with biotin, and hybridized to the chip for 16 hours at 49°C rotating at 60 rpm according to manufacturer's protocols (GeneChip CustomSeq Resequencing Array Protocol, Vers.2; Affymetrix, Santa Clara, CA). The arrays were subsequently washed and stained on a fluidics station followed by the scanning on a GeneChip 3000 Scanner (Affymetrix, Santa Clara, CA), and the raw data were analyzed using Affymetrix GeneChip Resequencing Analysis Software (GSEQ® v4.0) which enabled alignment of patients sequences against a reference sequence. All array data is MIAME compliant, and the raw data has been deposited in EBI Array Express database, a MIAME compliant database as detailed on the MGED Society website [18] under accession number E-MTAB-786.

**Figure 1:**
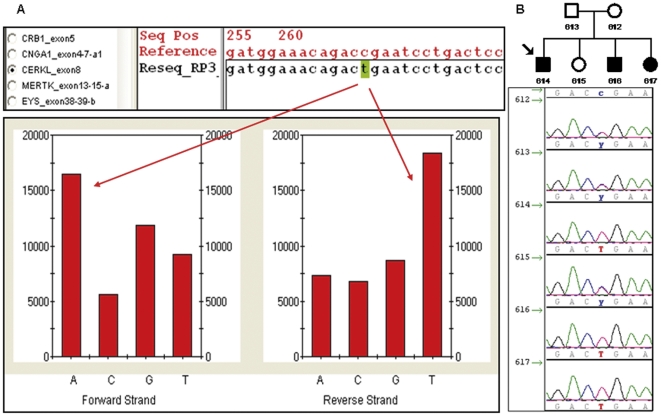
Analytic workflow. **A**. Genechip resequencing analysis software output for of the exon 8 of *CERKL* for 1 sample (index patient of the Family RP76) compared to reference sequence using GSEQ. The chip data reveals a nonsense mutation in *CERKL* (c.769C>T; p.R257X) at position 268 in the tiled sequence. The reference sequence carries a homozygous C in that position. The intensity histogram below shows how the mutant sample with a homozygous T binds most strongly to the probe with A on the forward strand and T on the reverse one. **B**. Electropherogram depiction of the members of the family RP76 confirming the co-segregation of the variant with the disease.

The novel identified variants were subsequently verified and screened in healthy controls by dideoxy sequencing and if additional family members were available, segregation of the variant with the disease was assessed (Figure 1).

In order to evaluate the pathogenicity of the novel variants, we analysed the potential impact of a given variant on the function or structure of the encoded protein based on conservation, physical properties of the amino acids, or in its possible occurrence in regulatory or splicing motifs using the software PupaSuite [19], [20]. To study the *EYS* variants, the domain architecture prediction and the alignment of the different orthologs were performed using bl2seq (NCBI) and EMBOSS Pairwise Alignment Algorithms: Needle and Water (EBI) alignments. The fully characterised SPAM proteins were aligned using MUSCLE (Multiple Sequence Comparison by Log-Expectation) program at EBI (For more details on the *EYS* bioinformatic characterization see [5]).

### MLPA Analysis of the *EYS* Gene

To clarify the molecular genetic cause in those families with just a single heterozygotic detected change in *EYS*, MLPA (Multiplex Ligation Probe Amplification) was performed to identify the second variant as an alternative approach to screen for copy number variations (CNVs), given that these type of mutations are particularly frequent in this gene [14]. Thus, 4 of the arRP patients were analysed by MLPA and gene dosage variations on *EYS* were evaluated as described elsewhere [14].

## Results

### Validation of the Array: Assessment of Positive Controls

The PCR of 3 out of the 93 amplicons (3.2%) could not be optimized; therefore, hybridization failed and these exons were excluded from further analysis. The average call rates for successfully hybridized amplicons were of 92% for the 102 arrays. Several algorithm parameters regarding this base calling can be altered, affecting the call rate and accuracy of the base calls [16]. The highest call rate was obtained using a Quality Score Threshold (QST) of 2 and without Base Reliability Threshold (BRT) [21]. Therefore, these settings were used for the call rate assessment. In addition, we observed a call rate constant improvement as the number of experiments increased. GSEQ contains a learning algorithm derived from ABACUS, an adaptative background genotype calling scheme to optimize data from multiple arrays analyzed together; larger batch sizes in an analysis are thus expected to have greater accuracy [22], [23]. The no-calls regions are mainly observed in G-C rich areas and repetitive elements unsuitable for analysis (ie, SINE, LINE, ALU, etc). *PDE6B* and *CERKL* were particularly rich in such unread nucleotides. So far, no arRP mutations have been reported in such regions [24].

To determine the ability of our chip to detect different types of mutations, we processed the DNA of 4 arRP patients carrying known mutations in 2 of the genes tiled onto the array (Table 1). The arrays used in this study were not designed to specifically identify deletions or insertions and, as expected, the insertion that was in a heterozygous state could not be detected by the GSEQ software. However, the substitution variants were detected.

### Variants Identified by the arRP Array

A total of 42 sequence changes were identified by the arRP arrays, of which 13 were potentially pathogenic variants affecting 14 out of the 98 patients (14%) (Table 2). Briefly, 5 of the 13 changes detected were known mutations comprising 1 nonsense substitution: p.R257X in *CERKL* (Figure 1) present in two unrelated families, 3 missense mutations: p.C948Y in *CRB1*, p.S297R and p.T342M in *RHO* and 1 acceptor site mutations: c.1297-2A>G in *MERTK*, whereas the other 8 potentially pathogenic variants were novel sequence changes: 6 missense and 2 splice site variants, all of them absent in control population. The potentially pathogenic variants were detected in 10 of the 16 retinal disease genes tiled on the array.

**Table 2:** Potentially pathogenic variants detected by the arRP Array and the MLPA.

Family ID	Gene	Nucleotide change	Amino acid change	Novel/Reference	Control population studies (mutant/normal alleles)	Genotype
RP 76	*CERKL*	c.769C>T	p.R257X	[34]	-	Homozygous
RP 206	*CERKL*	c.769C>T	p.R257X	[34]	-	Homozygous
RP 95	*CNGA1*	c.1151T>C	p.I384T	Novel†	0/200	Heterozygous
RP 29	*CRB1*	c.2843G>A	p.C948Y	[38]	-	Homozygous
RP 234	*EYS*	c.3695T>C	p.I1232T	Novel	0/200	Heterozygous
RP 234	*EYS*	c.1767-?_2023+?del	p.C590YfsX4	[26]	-	Heterozygous
RP 234	*EYS*	c.1971delT	p.S658VfsX4	[26]	-	Heterozygous
RP 109	*EYS*	c.5928-2A>G	-	Novel◊	0/200	Heterozygous
RP 202	*EYS*	c.8003G>T	p.C2668F	Novel*	0/200	Heterozygous
RP 96	*MERTK*	c.1297-2A>G	-	[39]	-	Homozygous
RP 353	*PROM1*	c.1532C>A	p.T520K	Novel	0/200	Heterozygous
RP 242	*RHO*	c.891C>T	p.S297R	[40]	-	Heterozygous
RP 322	*RHO*	c.1025C>T	p.T342M	[41]	-	Homozygous
RP 108	*RLBP1*	c.875A>T	p.T292M	Novel	0/200	Heterozygous
RP 193	*RPE65*	c.726-3C>A	-	Novel◊	0/200	Heterozygous
RP 333	*TULP1*	c.539G>A	p.R180H	Novel†	0/200	Heterozygous

*Disulfide Bond Alteration predicted by Dianna 1.1.

† Predicted as possibly damaging by Polyphen (V1).

◊ Splicing site Mutation by Berkeley Drosophila Genome Project (BDGP) website [42], [43].

Regarding the missense novel mutations, comparison between the orthologs from different species revealed a high level of conservation in 5 out of the 6 substituted residues: Ile384 in *CNGA1*, Ile1232 and Cys2668 in *EYS*, Thr292 in *RLBP1* and Arg180 in *TULP1* (Figure 3). In addition, bioinformatic tools employed to evaluate both the impact of the novel sequence changes on the transcription or translation mechanisms predicted that 2 of the missense variants were possibly damaging (p.I384T in *CNGA1* and p.R180H in *TULP1*), 2 of them were shown to abolish the splicing acceptor site (c.5928-2A>G in *EYS* and c.726-3C>A in *RPE65*), and 1 disulfide bond of the *EYS/*SPAM structure seemed to be compromised by the amino acidic substitution of a Cysteine to a Phenylalanine in the position 2668.

The 13 identified potentially pathogenic sequence changes were present in 14 out of the 98 patients included in this study. Of them, 5 patients were homozygous for the mutation and the 9 remainder were heterozygous. For patients with a single heterozygotic potentially pathogenic variant, further analyses of genetic variants not detectable by our customized resequencing chip would be valuable for the detection of the second mutation in arRP genes.

The remaining 29 detected sequence changes comprised 9 amino acid substitutions, 1 5′UTR variant, 6 synonymous changes, and 13 intronic variants that do not have any predicted deleterious effect on splicing, can be considered as unreported SNPs (Table S1). Among the novel nonsynonymous changes, p.G618S in *EYS* was initially reported as disease-causing variant [25], but the segregation studies results in the Spanish family discarded their pathogenic role. The detection of these SNPs is a good validation test for the array as it illustrates the ability of the chip to detect single base pair substitutions

### MLPA Analysis of the *EYS* Gene

**Figure 2:**
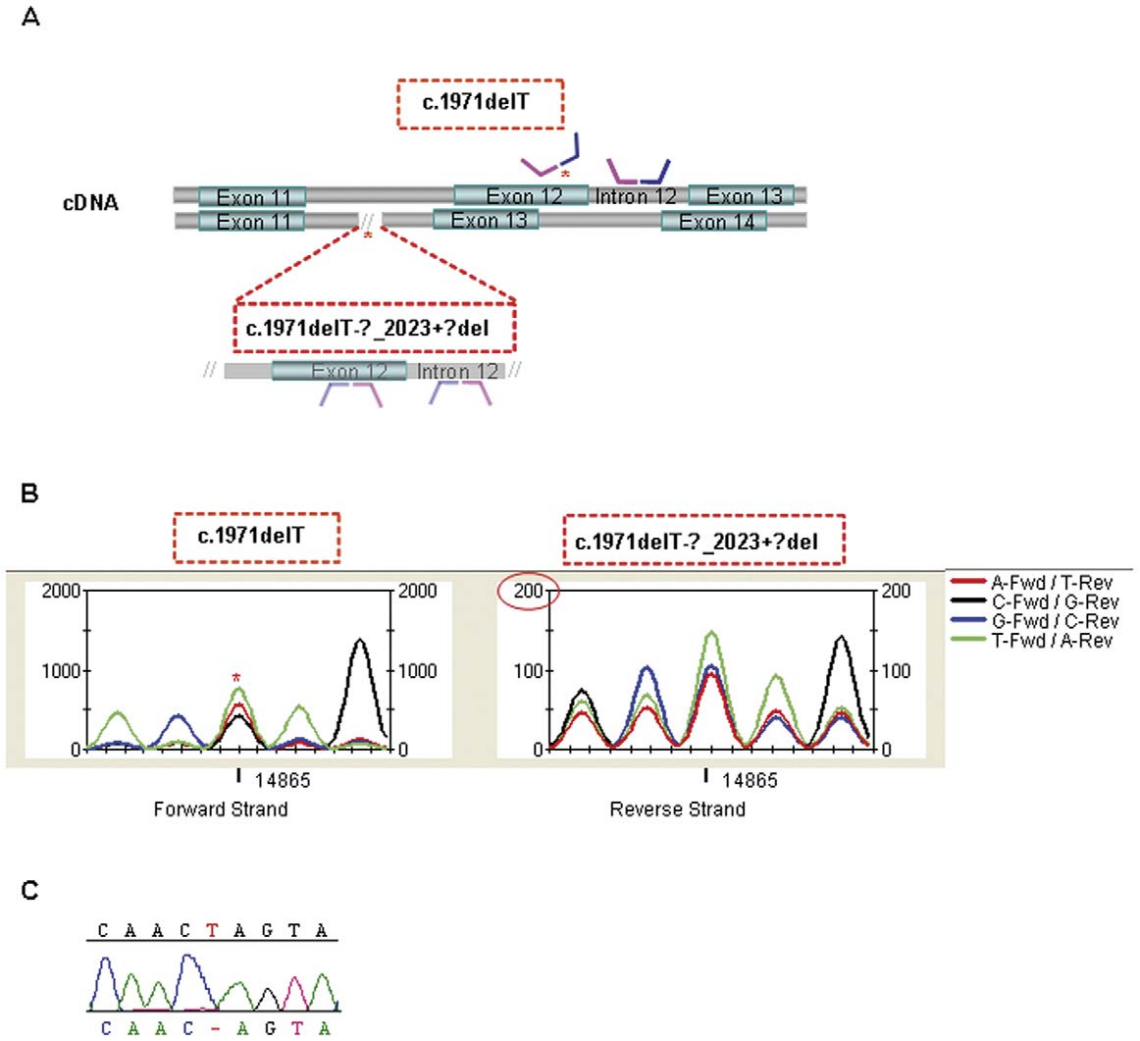
Mutations identified in the RP 234 family using several detection approaches. **A**. Schematic representation of the MLPA hybridization probe regions of *EYS* exons 11–14. The asterisks show the location of the mutations and how they affect the hybridization process. The MLPA dosage readouts and the fragment sizes for the exon-intron 12 are also represented in a box. **B**. Resequencing trace graph of the five bases interval including the mutated single nucleotide (c.1971delT). Forward and reverse strands correspond to each of the patient alleles. Manual examination of signal intensity data (Y axis) is coherent with the two mutations. The asterisk points the deleted base in the forward allele. The intensity in reverse strand is 10-fold lower than forward (circle in red) suggesting that this allele is deleted (c.1767-?_2023+?del). **C**. Dideoxy sequencing electropherogram of exon 12 of the index patient confirming the deletion of a T (c.1971delT).

The present study led to the identification of one intragenic *EYS* rearrangement in the family RP 234 that carries a novel very likely pathogenic change (p.I1232T, absent in controls). MLPA analysis revealed 0% dosage in exon 12 of *EYS* whereas 50% dosage was shown in the adjacent intron 12. The resequencing data of that exon showed an apparently normal readout suggesting that at least one allele is present. This situation could be explained by the co-existence of two independent pathogenic events occurring in different alleles. One of them is the heterozygous deletion of the exon12-intron12, and the other one would be an underlying defect on the DNA sequence where the MLPA probe should hybrid. As we expected, further dideoxy sequencing showed a homozygous deletion of 1 bp disabling the correct hybridization of the MLPA probe (Figure 2). It is noteworthy that both the large deletion (c.1767-?_2023+?del; p.C590YfsX4) and the 1 bp deletion (c.1971delT; p.S658VfsX4) are known mutations previously described by our group in other unrelated RP families [14], [26] (Table 2).

**Figure 3:**
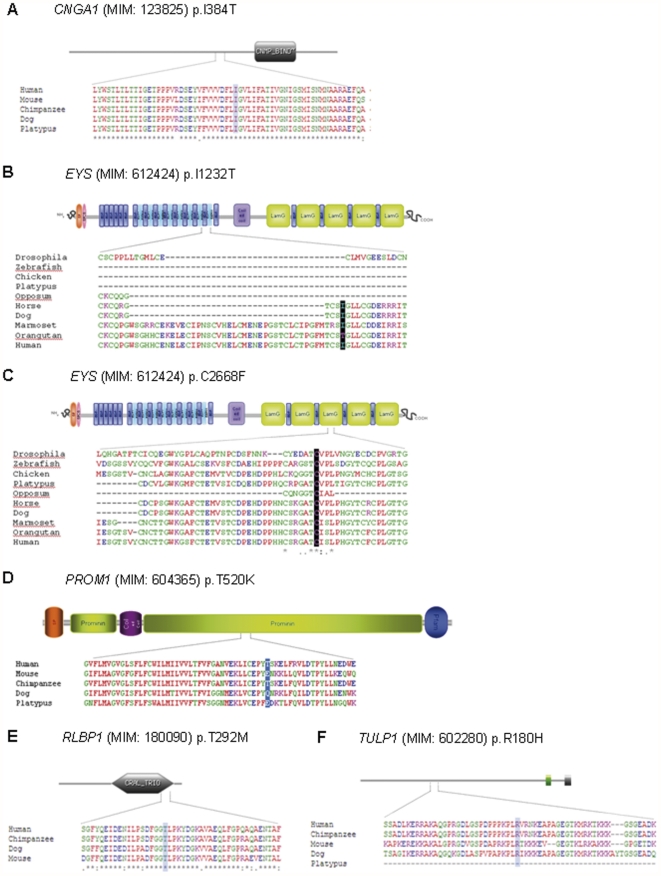
ScanProsite predicted domains of the proteins and ClustalW alignment of the orthologs from different species. **A**. *CNGA1* (p.I384T). **B**. *EYS* (p.I1232T). **C**. *EYS* (p.C2668F). **D**. *PROM1* (p.T520K). **E**. *RLBP1* (p.T292M). **F**. *TULP1* (p.R180H).To study the *EYS* variants, we have used the orthologs alignment performed in Barragan *et al*. [5]. The residue highlighted is mutated.

In an attempt to define a characteristic profile that may detect these heterozygous mutations by our resequencing approach, the intensity signal of each strand was evaluated. Forward and reverse strands correspond to each of the patient alleles. The results of the manual interrogation were coherent with the two mutated alleles. The deleted T appeared as an unclear nucleotide in the forward strand whereas the deletion of the whole allele manifested as a drop in the intensity signal in the reverse strand (Figure 2).

## Discussion

Molecular diagnosis of RP is a challenging task given the huge genetic heterogeneity of this disease. The large number of deleterious mutations that can be found in each gene, and the multiple genes that can be mutated to give the same phenotype, make the detection of mutations by traditional screening methods costly and time consuming. Thus, there is an urgent need for a validated screening method that allows the detection of mutations simultaneously in several genes in a single high-throughput platform. Microarray sequencing technology offers a rapid method for detecting mutations in patients with genetically heterogeneous diseases such as arRP. Using the resequencing technology to read comprehensive nucleotide sequence of a number of genes presents some advantages in comparison with other available techniques. The resequencing chip is 5 to 10 times less expensive than conventional sequencing [12] and although the APEX array is cheaper, the resequencing chip provides the significant benefit of detecting novel variants.

The detection of known and novel mutations in this study establishes array-based resequencing as an effective tool with potential to improve diagnosis, which hopefully may help to provide genetic counselling and give a more reliable prognosis in patients and their families.

Mutational screening of arRP patients using resequencing array-based technology has been previously reported [11]–[13], but our arRP sequencing array offers for the first time an opportunity to screen for sequence alterations in the *EYS* gene. The high number of *EYS* mutations detected by PCR based direct dideoxy genomic sequencing published in different arRP patients and the diverse ethnic origins of these families [5], [25]–[30] set this gene as the most prevalent one in arRP (15.9% in Spanish families) [5]. The combination of the large size of this gene and the lack of hot spots of disease-causing mutations make the screening of this gene using traditional methods slow and expensive, but these disadvantages can now be overcome by the implementation of the array-based resequencing technology.

The call rates for the arrays in this study (92%) are within the range of previous studies (90–99%). Considering that this study utilised higher numbers of arrays than previous studies, we would have expected the call rates to be higher but the main problem here was due to hybridization failures resulting in no-called regions. Certain probes as those with higher than average GC content or those containing repeat regions can be problematic for resequencing arrays and are more likely to be no-called. Analysis of the sequence on RepeatMasker revealed that some regions of the genes *CNGA1*, *TULP1* and *PDE6B* are particularly rich in repeat sequences resulting in decreased signal and increased chance of being no-called. Extreme caution must be exerted in designing the probes content of the chip in order to avoid a call rate decrease.

The arRP chip reported herein can screen for most known disease causing mutations due to single nucleotide changes but as expected, heterozygous deletions, insertions and CNVs have been the main problem for the array. In part, some of these limitations can be overcome with improvements in the chip design like including specific probes for known insertions and deletions [21], [31], [32] but they only allow the detection of known changes and it would be convenient to periodically update the resequencing design with newer insertions and deletions. For that reason and for the high prevalence of CNVs recently reported in the *EYS* gene in Spanish families [14], we decided to use the MLPA technology in those families where only one pathogenic change had been identified by the resequencing approach in that gene. Despite the reduced number of families included on the MLPA study, the results obtained were very interesting and allowed the identification of two independent pathogenic events in one patient (Family RP 234) affecting two different alleles. These results evidenced that the MLPA technique is able to detect not only CNVs but also short deletions of 1 bp if they are located within the hybridization region of the probe. In our opinion, the combination of the two screening strategies is currently the most rapid and efficient method for mutation screening of arRP in clinical practice. Regarding the family RP 234, a third heterozygous variant was detected in the *EYS* gene (c.3695T>C; p.I1232T) absent in controls and evolutionarily conserved. Possibly, this variation could represent a rare SNP but also a mutation that may modify the phenotype. Such mechanisms involving the presence of modifier alleles have been suggested to explain variability in disease phenotype among affected family members with retinal dystrophies [33]. Great caution must be exerted when interpreting the functional effect of such novel variants and a more comprehensive study in additional members of the family would reveal them to be disease causing, modifier or polymorphic variants.

In addition to mutations detected in the genes routinely tested for arRP, we identified two mutations (p.T342M and p.S297R) typically associated with autosomal dominant cases of RP (adRP) in the rhodopsine gene, *Rho*. This evidenced that *Rho* not only plays an important role in the pathogenesis of adRP, but it is also involved in a number of arRP cases and its routine analysis in those families should be considered.

Most of the RP associated genes have hundreds of disease alleles but sometimes one predominates. A few examples are the deleterious *c.769C>T* allele of *CERKL* that causes a substantial proportion of sporadic and arRP Spanish cases [34], [35], and the *c.2688T>A* allele of *CRB1* described as a frequent mutation by Vallespin *et al*. [36]. The *EYS* variants *c.1971delT* and *c.1767-?_2023+?del* have also been detected in unrelated Spanish and French families [14], [26]. Yet haplotype analyses would be valuable to determine the origin of these mutations, identifying recurrent mutations in Caucasian and especially specific populations such as the Spanish one provides an essential source for the molecular and clinical diagnosis of such a heterogeneous disease.

Although further refinements in array design, analysis algorithms or both would need to be performed to improve this tool and optimise research translation into the clinical setting, this work shows that resequencing array-based technology can be used as a rapid screening tool. The large amount of data generated by this high-throughput methodology is an estimable resource not only for the establishment of genotype-phenotype correlations, but also for the identification of modifier alleles that could be responsible of the significant phenotypic variability of RP. Furthermore, this technology has provided new information and enhances our understanding of the aetiology and pathogenesis of such as genetically heterogeneous disease, and ultimately may lead to better clinical management of patients and their families.

Finally, the design of this array can easily be transformed in an equivalent diagnostic system based on targeted enrichment followed by next generation sequencing.

## Supporting Information

Table S1 Unlikely but unknown pathogenic coding variants detected by the arrp array.
(DOC)
