# Supplementary material for: Mutation Screening of Multiple Genes in Spanish Patients with Autosomal Recessive Retinitis Pigmentosa by Targeted Resequencing
Source: PLoS One. 2011 Dec 2;6(12):e27894. doi: 10.1371/journal.pone.0027894 (PMC3229495; doi:10.1371/journal.pone.0027894)
Supplement: Table S1 — Unlikely but unknown pathogenic coding variants detected by the arrp array. (DOC) [file pone.0027894.s001.doc]

**Supplementary Table 1. Unlikely but unknown pathogenic coding variants detected by the arRP Array.**

| **Gene** | **Nucleotide change** | **Amino acid change** | **Genotype** | **Frequency** | **Control alleles (mutant/**  **normal alleles)** | **Family segregation results** | **Conclusions** |
| --- | --- | --- | --- | --- | --- | --- | --- |
| *CNGA1* | c.372C>T | p.N124N | Heterozygous | 3 patients | N/A | Does not segregate | Synonymous |
| *CNGA1* | c.495T>C | p.Y165Y | Heterozygous | 1 patient | N/A | N/A | Synonymous |
| *CNGA1* | c.1834A>C | p.N612H | Heterozygous | 1 patient | N/A | Does not segregate | Polymorphic amino acid substitution |
| *CRB1* | c.135C>G | p.C45W | Heterozygous | 1 patient | N/A | Does not segregate | Polymorphic amino acid substitution |
| *CRB1* | c.3886A>C | p.K1296Q | Heterozygous and Homozygous | 12 patients | N/A | Does not segregate | Polymorphic amino acid substitution |
| *CRB1* | c.3960G>C | p.Q1320H | Heterozygous | 1 patient | N/A | Does not segregate | Polymorphic amino acid substitution |
| *EYS* | c.-459C>T | - | Heterozygous | 1 patient | 20/200 |  | 5' UTR variant |
| *EYS* | c.1852G>A | p.G618S* | Heterozygous | 1 patient | N/A | Does not segregate | Polymorphic amino acid substitution |
| *EYS* | c.2024-14C>T | - | Homozygous | 1 patient | N/A | N/A | Predicted to do not affect the splicing |
| *EYS* | c.7228+7G>A | - | Heterozygous | 1 patient | N/A | N/A | Predicted to do not affect the splicing |
| *EYS* | c.7608C>T | p.I2536I | Heterozygous | 1 patient | N/A | N/A | Synonymous |
| *MERTK* | c.482+698T>C | - | Heterozygous | 1 patient | N/A | N/A | Predicted to do not affect the splicing |
| *MERTK* | c.482+921G>C | - | Heterozygous | 1 patient | N/A | N/A | Predicted to do not affect the splicing |
| *MERTK* | c.710G>A | p.R237H | Heterozygous | 1 patient | N/A | Does not segregate | Polymorphic amino acid substitution |
| *MERTK* | c.2349+15T>A | - | Heterozygous | 1 patient | N/A | N/A | Predicted to do not affect the splicing |
| *MERTK* | c.2350-15T>C | - | Heterozygous | 1 patient | N/A | N/A | Predicted to affect slightly the splicing |
| *NR2E3* | c.197G>C | p.G66A | Heterozygous | 4 patients | N/A | Does not segregate | Polymorphic amino acid substitution |
| *PDE6B* | c.615C>T | p.D205D | Heterozygous | 1 patient | N/A | N/A | Synonymous |
| *PROM1* | c.55T>G | p.S19A | Heterozygous | 2 patients | 2/200 | Does not segregate | Polymorphic amino acid substitution |
| *PROM1* | c.1470C>T | p.I500I | Heterozygous | 1 patient | N/A | N/A | Synonymous |
| *PROM1* | c.1741-5C>T | - | Heterozygous | 3 patients | N/A | N/A | Predicted to do not affect the splicing |
| *PROM1* | c.2050-15T>C | - | Heterozygous | 2 patients | N/A | N/A | Predicted to do not affect the Splicing |
| *PROM1* | c.2347-3T>C | - | Heterozygous | 3 patients | N/A | N/A | Predicted to do not affect the Splicing |
| *RHO* | c.444C>T | p.F148F | Heterozygous | 1 patient | N/A | N/A | Synonymous |
| *RHO* | c.502G>A | p.A168T | Heterozygous | 1 patient | N/A | Does not segregate | Polymorphic amino acid substitution |
| *RGR* | c.756+5A>G | - | Heterozygous | 1 patient | N/A | N/A | Predicted to do not affect the Splicing |
| *RPE65* | c.1243+45T>G | - | Heterozygous | 1 patient | N/A | N/A | Predicted to do not affect the Splicing |
| *RPE65* | c.1339-48A>G | - | Heterozygous | 1 patient | N/A | N/A | Predicted to do not affect the Splicing |
| *TULP1* | c.1224+26C>T | - | Heterozygous | 1 patient | N/A | N/A | Predicted to do not affect the Splicing |

* p.G618S previously reported by Audo *et al* [25].

N/A: Not available
